# Improving management of ARDS: uniting acute management and long-term recovery

**DOI:** 10.1186/s13054-024-04810-9

**Published:** 2024-02-23

**Authors:** Nicola Latronico, M. Eikermann, E. W. Ely, D. M. Needham

**Affiliations:** 1 Department of Medical and Surgical Specialties, Radiological Sciences and Public Health, University of Brescia, Brescia, Italy; 2 Department of Emergency, Spedali Civili University Hospital, Piazzale Ospedali Civili, 1, 25123 Brescia, Italy; 3 “Alessandra BONO” Interdepartmental University Research Center on Long-Term Outcome (LOTO) in Critical Illness Survivors, University of Brescia, Brescia, Italy; 4 Department of Anesthesiology, Albert Einstein College of Medicine, Montefiore Medical Center, New York, USA; 5 Klinik fur Anästhesiologie und Intensivmedizin, Universitaet Duisburg-Essen, Essen, Germany; 6 Department of Medicine, Division of Allergy, Pulmonary and Critical Care Medicine, Critical Illness, Brain Dysfunction, and Survivorship (CIBS) Center, Vanderbilt University Medical Center, Nashville, TN USA; 7 Tennessee Valley Veteran’s Affairs Geriatric Research Education Clinical Center, VA Tennessee Valley Healthcare System, Nashville, TN USA; 8 Outcomes After Critical Illness and Surgery (OACIS) Group, Johns Hopkins University, Baltimore, MD USA; 9 Division of Pulmonary and Critical Care Medicine, Department of Medicine, Johns Hopkins University School of Medicine, Baltimore, MD USA; 10 Department of Physical Medicine and Rehabilitation, Johns Hopkins University School of Medicine, Baltimore, MD USA; 11 Johns Hopkins University School of Nursing, Baltimore, MD USA

**Keywords:** ARDS, Long-term outcome, Chronic disability, Post-intensive care syndrome

## Abstract

Acute Respiratory Distress Syndrome (ARDS) is an important global health issue with high in-hospital mortality. Importantly, the impact of ARDS extends beyond the acute phase, with increased mortality and disability for months to years after hospitalization. These findings underscore the importance of extended follow-up to assess and address the Post-Intensive Care Syndrome (PICS), characterized by persistent impairments in physical, cognitive, and/or mental health status that impair quality of life over the long-term. Persistent muscle weakness is a common physical problem for ARDS survivors, affecting mobility and activities of daily living. Critical illness and related interventions, including prolonged bed rest and overuse of sedatives and neuromuscular blocking agents during mechanical ventilation, are important risk factors for ICU-acquired weakness. Deep sedation also increases the risk of delirium in the ICU, and long-term cognitive impairment. Corticosteroids also may be used during management of ARDS, particularly in the setting of COVID-19. Corticosteroids can be associated with myopathy and muscle weakness, as well as prolonged delirium that increases the risk of long-term cognitive impairment. The optimal duration and dosage of corticosteroids remain uncertain, and there's limited long-term data on their effects on muscle weakness and cognition in ARDS survivors. In addition to physical and cognitive issues, mental health challenges, such as depression, anxiety, and post-traumatic stress disorder, are common in ARDS survivors. Strategies to address these complications emphasize the need for consistent implementation of the evidence-based ABCDEF bundle, which includes daily management of analgesia in concert with early cessation of sedatives, avoidance of benzodiazepines, daily delirium monitoring and management, early mobilization, and incorporation of family at the bedside. In conclusion, ARDS is a complex global health challenge with consequences extending beyond the acute phase. Understanding the links between critical care management and long-term consequences is vital for developing effective therapeutic strategies and improving the quality of life for ARDS survivors.

## Background

Acute respiratory distress syndrome (ARDS) is a major health problem worldwide. Despite improvements in supportive treatment, in-hospital mortality remains high at approximately 40% [1]. There is great variability and important differences in patients management across geo-economic regions, with treatments of proven efficacy for reducing mortality, such as low tidal volume ventilation, not consistently implemented [2]. ARDS is a clinically and biologically heterogeneous condition, implying that patients may respond differently to therapeutic interventions. Interventions that are effective in wealthy countries where the population is predominantly older and of European descent may not be effective in other settings where patients are young and have different patterns of comorbidities and risk factors [3]. Longer-term mortality remains high for months to years after surviving initial hospitalization for ARDS, particularly in older patients, with nearly 25% absolute increase in late mortality compared to non-hospitalized patients [4], underscoring the need for prolonged follow-up of ARDS survivors. Prolonged follow-up is even more important in the evaluation of the post-intensive care syndrome (PICS), which is conventionally described as new or worsening impairments in physical, cognitive, or mental health status arising in subjects surviving critical illness and persisting beyond acute care hospitalization [5]. Importantly, short and long-term mortality and disability are the consequence of both the severity of ARDS and iatrogenic and disease-related complications that should be prevented or treated in the earliest stages of ICU care. In this Perspective, we focus on the relationship between the acute stage of ARDS and long-term disability, and suggest effective therapeutic strategies.

## Main text

Physical dysfunction in survivors of critical illness include a wide range of impairments at the level of body function and structure, and for the whole person [6]. Severe muscle weakness, defined as ICU-acquired paresis [7] or weakness [8] or critical illness weakness (CIW) [9], is the most frequently reported long-term physical impairment in ARDS survivors. More than one-third of patients with ARDS has muscle weakness that prevents them from walking and being independent in activities of daily living at the time of hospital discharge [10]. At 2 years, 9% of patients are still weak, and an even greater proportion are unable to walk quickly and have a significantly impaired self- perception of their physical condition [10]. After 5 years, patients still report muscle weakness, even if not detected with routine physical examination, and demonstrate a 24% reduction in 6-min walk distance compared with an age- and sex-matched control population [11].

**Fig. 1:**
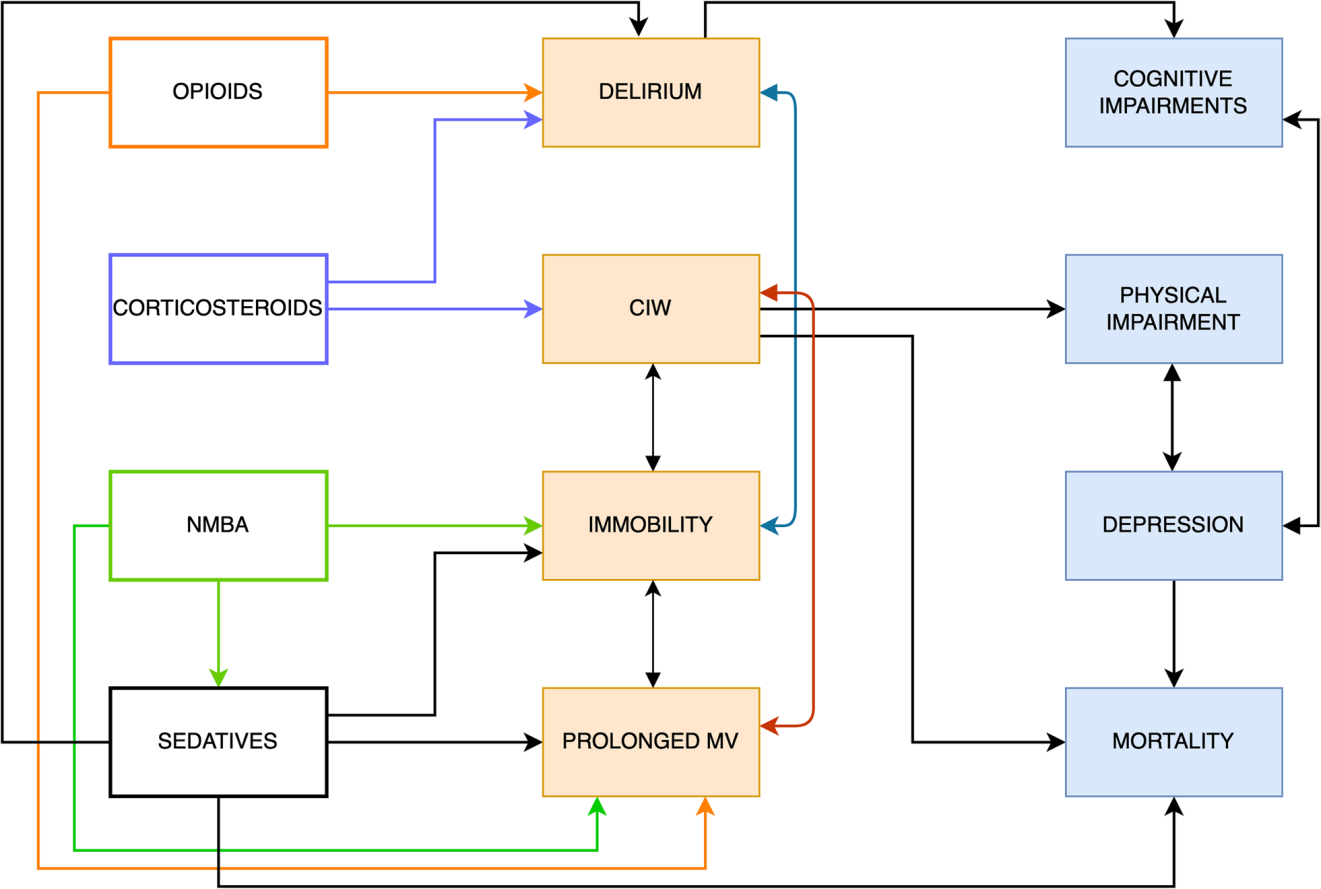
Relationship between aspects of acute ARDS management, critical illness-associated sequelae and long-term mortality and morbidity. The four classes of drugs considered (left part of the figure) are important treatments in patients with ARDS but are also relevant risk factors for the development of ICU complications (middle). These “iatrogenic” complications are strongly associated with long-term morbidity and mortality (right)

Immobility is a powerful predictor and/or cause of CIW [10], which in turn is associated with prolonged mechanical ventilation, and an increased risk of short- and long-term mortality and persistent physical disability [12, 13] (Fig. 1). As an example of an iatrogenic cause of immobility, continuous NMBA infusion is used to reduce the work of breathing and patient-ventilator dyssynchrony in mechanically ventilated patients; however, NMBA infusion inevitably causes immobilization and requires deep sedation, both of which may adversely affect mortality [14, 15]. As a result, the net effect of NMBA on patient outcomes remains uncertain and its routine use is not recommended in ARDS [16]. Meta-analysis showed only a modest association between NMBA and severe weakness, but this finding was only based on cohort studies with high heterogeneity allowing a low level of certainty. In addition, these studies included mainly patients with sepsis or septic shock. Four randomized controlled trials focused on patients with ARDS, all with important limitations concerning the diagnosis of CIW that was based on clinical assessment alone without electromyographic investigations or done early during ICU stay [17]. The ROSE trial, which randomized ARDS patients to 48 h of continuous NMBA infusion and deep sedation versus usual care without routine NMBA infusion and with lighter sedation, found no significant differences in mortality at 3 months. Notably, muscle strength measured using the Medical Research Council scale could not be assessed in 51–67% of patients during the hospital stay and was not assessed beyond hospital discharge. There is much to continue learning regarding NMBA infusion and patient outcomes.

As another example, corticosteroids are a standard treatment in COVID-19 ARDS [18] and may reduce mortality in non-COVID-19 ARDS [19, 20]. However, corticosteroids also are a risk factor for severe muscle weakness and physical dysfunction [21], particularly if used in conjunction with NMBA [22]. Additionally, the use of corticosteroids is significantly associated with transitioning to delirium from a non-delirious state, [23] thus increasing the risk of developing cognitive impairment after hospital discharge [24]. Similar harms have been documented with the use of opioids in the ICU, which is associated with an increased risk of delirium in a dose-dependent manner [25]. Identifying the optimal treatment duration and dose of commonly-used medications, such as corticosteroids, NMBA, opioids, sedatives, vasoactive medications (norepinephrine, vasopressin, phenylephrine), aminoglycosides and vancomycin would be of great importance to helping optimize ARDS survivors’ outcomes [21, 26, 27].

Stress hyperglycemia is an example of a disease-related complication that is worsened by corticosteroid use, and is a risk factor for critical illness polyneuropathy (and hence, for muscle weakness) and incident diabetes [28]. By avoiding hyperglycemia, muscle weakness may be reduced [28]. In a recent large randomized trial evaluating tight blood glucose control without early parenteral nutrition, hyperglycemia was significantly less common compared to early use of parenteral nutrition [29]. However, muscle weakness and long-term outcomes were not reported in this trial and remain important areas of investigation.

Mental health outcomes, such as symptoms of depression, severe anxiety, and post-traumatic stress disorder, are highly prevalent in ARDS survivors [30]. Half of ARDS survivors experience psychological symptoms for months or years after discharge, and nearly one-third report symptoms of depression, anxiety and post-traumatic stress disorder that are often co-occurring [31]. Depression has an impact on mortality and new impairments in physical functioning, with potential to create worse depression symptoms [30, 32, 33]. Increased risk of self-harm and suicide are among the most severe consequences of such mental health impairments in ICU survivors [34].

Delirium, a depersonalizing condition that is associated with long-term cognitive impairment [24, 35], is a major concern in ARDS. Delirium not only disconnects the patients cognitively from everyone around them at perhaps the most vulnerable period of time in his or her life, but also distances caregivers from patients, which in a way severs the healing connection we so desire. Delirium is not inevitable in ARDS, and it’s prevalence and duration must be mitigated. Strategies to reduce delirium are mainly nonpharmacological, emphasizing: (a) early mobilization and rehabilitation, which may also reduce the duration of mechanical ventilation [36] and improve long-term cognitive impairment [37]; (b) replacing routine use of infusions of sedation medications with patient-centered symptom control [38]; and (c) the “ABCDEF” bundle of evidence-based practices that serve to bolster the art and practice of humanism in medicine [39, 40].

## Conclusions

There is a *close link* between complications arising during the acute stage of ARDS and long-term outcomes in survivors. This has two important implications. First, improving the long-term outcome of patients with ARDS also comes through the meticulous prevention and rapid treatment of these complications. Important, in the last 50 years we have learnt that “attention to the little things early in a patient’s critical illness makes the greatest difference in outcomes” [41]. We must recognize that survivorship care begins early during acute stage of ARDS, ideally on the first day the patient is admitted to the ICU [42]. As treatment-related complications are multiple and interrelated, it will be important to define optimal treatment ranges and monitoring methods. Second, assessment of long-term outcomes in ARDS survivors (indeed in all ICU survivors) must be a top priority on the ICU agenda. We suggest that the ‘A’ in ARDS should serve as a mental reminder that ‘After’ hospital discharge, following ARDS, high priority should be given to the diagnosis, prevention and treatment of all aspects of PICS [43] with further evaluation of the role of ICU-recovery or follow-up centers [9, 44, 45].

Future recommendations to improve the outcome of patients with ARDS should be devoted to the entire patient journey from ICU admission to return home, and should be developed by key professional associations involved in critical care, mental health/neuro-psychology and rehabilitation along with survivors and their family members, as was done more than 10 years ago in the United States [5, 46]. Because ARDS is a global problem, it is essential that participants are representative of the geographic, economic, and social context in which ARDS develops and that long-term outcome measures are adjusted to be useful also in low-income and middle-income areas. Such work would be a strategic investment to better understand modifiable acute-care risk factors (including iatrogenic factors) and the most effective treatments to improve long-term patient-centered outcomes worldwide.

## Data Availability

Not applicable.
